# Four Wave Mixing control in a photonic molecule made by silicon microring resonators

**DOI:** 10.1038/s41598-018-36694-5

**Published:** 2019-01-23

**Authors:** Massimo Borghi, Alessandro Trenti, Lorenzo Pavesi

**Affiliations:** 1 Nanoscience Laboratory, Department of Physics, University of Trento, I-38123 Povo, Italy; 2 Present Address: Quantum Engineering Technology Labs, H. H. Wills Physics Laboratory and Department of Electrical and Electronic Engineering, University of Bristol, Bristol, BS8 1FD UK; 3 Present Address: Vienna Center for Quantum Science and Technology (VCQ), Faculty of Physics, University of Vienna, Boltzmanngasse 5, 1090 Vienna, Austria

## Abstract

Four Wave Mixing (FWM) is the main nonlinear interaction in integrated silicon devices, which finds diffuse use in all-optical signal processing and wavelength conversion. Despite the numerous works on coupled resonator devices, which showed record conversion efficiencies and broadband operation, the possibility to coherently control the strength of the stimulated FWM interaction on a chip has received very limited attention. Here, we demonstrate both theoretically and experimentally, the manipulation of FWM in a photonic molecule based on two side coupled silicon microring resonators. The active tuning of the inter-resonator phase and of their eigenfrequencies allows setting the molecule in a sub-radiant state, where FWM is enhanced with respect to the isolated resonators. On the other hand, we can reconfigure the state of the photonic molecule to have energy equipartition among the resonators, and suppress FWM by making the two Signal waves to interfere destructively in the side coupled waveguides. This work constitutes an experimental demonstration of the control of a nonlinear parametric interaction via coherent oscillation phenomena in an integrated optical device.

## Introduction

Stimulated Four Wave Mixing (FWM), that is the all-optical, coherent energy transfer of a Signal wave into an Idler wave by means of two auxiliary Pump waves^1^, has been extensively studied for all-optical signal processing^2,3^, wavelength conversion^4,5^, frequency comb generation^6,7^, parametric oscillation and amplification^8,9^. To overcome the intrinsic weak *χ*^(3)^ nonlinearity of silicon and silicon based materials^1^, and/or to decrease the optical power required for efficient FWM, several strategies have been adopted. Slow light waveguides enhance the effective Kerr nonlinearity by a factor *S*^4^ (here *S* denotes the slowing factor) with respect to a bare waveguide^10^. Typically, these are realized with line-defect Photonic Crystals (PhC) waveguides, where the reduced group velocity, combined with the extremely small mode area, increases the nonlinear coefficient^11–15^. Another method exploits the internal Field Enhancement (FE) of optical resonators. Indeed, with respect to a waveguide, these systems have a FWM efficiency which scales as FE^8^ ^16^. Slow light waveguides, based on a cascade of *N* optical resonators, have been also demonstrated^10^. These have a FWM efficiency which scales as *N*^2^ with respect to a single cavity. Enhanced FWM through Coupled Resonators Optical Waveguides (CROW) has been shown with directly coupled microrings^17,18^ and PhC nanocavities^19,20^. Typically, these structures are treated as a whole, with tens or hundreds of repeating units. Long-range periodicity is deliberately sought to tailor the frequency-wavevector band diagram, in order to increase the group index while keeping the group velocity dispersion reasonably low^21^. However, dealing with a large number of unit cells inherently precludes the study of the impact on FWM of the inter-resonator phase and resonator eigenfrequencies relative detuning. Furthermore, these works are all focused on the enhancing of the parametric interaction, while little attention has been paid to explore the plenty of FWM regimes enabled by the structural complexity. In some works, photonic molecules^22,23^, constituted by two or three coupled resonators, have been analyzed in terms of their inter-cavity distance or their eigenfrequency separation, for the dynamical tuning of the Electromagnetic Induced Transparency (EIT) effect^24–28^, as well as for the onset of coherent collective phenomena like super or sub-radiance^29^. These studies were principally limited to a linear analysis, since their goal was mainly focused to slow-light or routing applications. Nonlinearities have been induced in these structures for light stopping^30^, storage^31^, cavity QED^32^, and spontaneous mirror-symmetry breaking^33^. The engineering of the field distribution inside photonic molecules has been exploited for FWM among orthogonal supermodes^34^ and for the dynamical tuning of the evanescent coupling between two different cavities^35,36^.

In this work, we investigate FWM in a system made by two silicon microring resonators (photonic dimer) which are side coupled by means of two waveguides. We aim at studying the coherent control of FWM and not to demonstrate record conversion efficiencies. We independently thermally tune the inter-resonator phase *ϕ*, and resonator eigenfrequency difference *δ*. We experimentally and theoretically demonstrate that, in the parameter space (*ϕ*,*δ*), the efficiency of FWM can be enhanced, left unchanged or completely suppressed with respect to the one of a single isolated resonator. These regimes cannot be easily resolved and accomplished in large structures, where the structural periodicity makes slow light effects to overwhelm any other side effect. Here, a FWM enhancement of (7.0 ± 0.2) dB with respect to each single constituent of the molecule is demonstrated. This efficiency increase is attributed to a sharp raise of the internal field enhancement of one of the resonator, caused by the presence of the other. We theoretically prove that this phenomenon is linked to the excitation of a sub-radiant mode of the photonic molecule. On the other hand, FWM suppression arises from the coherent destructive interference between the Signal waves which are generated in the two resonators and, then, coupled into the common side waveguide where they do interfere. We do experimentally map the energy distribution between the two resonators by monitoring the surface light scattered during the onset of the different FWM regimes, and further validate our comprehension of the phenomenon through an analysis performed in terms of the supermode of the structure.

## Results

### Theory of Stimulated Four Wave Mixing in two side coupled resonators

**Figure 1:**
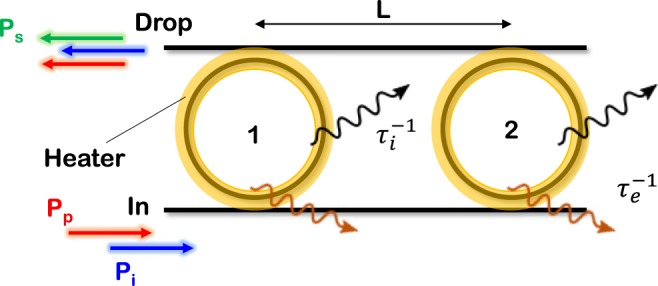
Sketch of the device under test. Two ring resonators, labelled 1 and 2, are separated by a distance *L* (center to center), and are both coupled to two side bus waveguides through a narrow coupling gap. The field decay rate *γ*_*e*_ in the waveguide is related to the extrinsic photon lifetime *τ*_*e*_ by *γ*_*e*_ = 1/*τ*_*e*_. Similarly, the field decay rate *γ*_*i*_ associated to intrinsic losses of the material is related to the intrinsic photon lifetime *τ*_*i*_ by *γ*_*i*_ = 1/*τ*_*i*_. Metallic microheaters (sketched in yellow) are placed on the top of each ring. The Pump (*P*_*p*_) and the Idler (*P*_*i*_) fields are injected into the In port, and are collected, together with the generated Signal (*P*_*s*_) by stimulated FWM, at the output of the Drop port.

In the following, the theoretical formalism which describes stimulated FWM in the photonic molecule made by two ring resonators is presented. We point out that even if the theory of FWM in similar coupled resonator structures like CROWs has been already reported^18^, it has been always carried out for perfect periodic structures and under resonant excitation. As a result, the FWM efficiency turned out to be dependent only on the number of resonators and on their quality factor, but not on their relative eigenfrequency detuning. Here we account also for this possibility, so as the overall FWM efficiency will depend on a larger set of degrees of freedom. The modeled structure, shown in Fig. 1, consists of two rings which have equal intrinsic photon lifetime *τ*_*i*_ and equal energy decay rates *γ*_*e*_ = 1/*τ*_*e*_ into the bus waveguides.

The set of Pump, Signal and Idler resonances involved in the FWM process will be labelled as *ω*_1(2),*j*_, where *j* = *p*, *s*, *i* and 1(2) refers to the ring. The structure is excited at the input port with two Continuous Wave (CW) lasers, with frequency *ω*_*p*_ and *ω*_*i*_, which can be slightly detuned from the two resonators eigenfrequencies by the quantities Δ*ω*_1(2),*p*_ and Δ*ω*_1(2),*i*_ respectively. The two lasers carry a power *P*_*p*_ = |*p*_*p*_|^2^ and *P*_*i*_ = |*p*_*i*_|^2^ respectively.

The coupled mode equations for the slowly varying energy envelopes *a*_*i*,*p*,*s*_ in the two resonators are given by^37^:

1 $$\begin{array}{rcl}\frac{d{a}_{1,p(i)}}{dt} & = & i({\omega }_{1,p(i)}-{\omega }_{p(i)}+\frac{i}{{\tau }_{{\rm{tot}}}}){a}_{1,p(i)}-\mu {a}_{2,p(i)}+i\sqrt{\frac{2}{{\tau }_{e}}}{p}_{p(i)}\\ \frac{d{a}_{2,p(i)}}{dt} & = & i({\omega }_{2,p(i)}-{\omega }_{p(i)}+\frac{i}{{\tau }_{{\rm{tot}}}}){a}_{2,p(i)}-\mu {a}_{1,p(i)}+i\sqrt{\frac{2}{{\tau }_{e}}}{e}^{-i{\varphi }_{p(i)}}{p}_{p(i)}\\ \frac{d{a}_{1,s}}{dt} & = & i({\omega }_{1,s}-{\omega }_{s}+\frac{i}{{\tau }_{{\rm{tot}}}}){a}_{1,s}-\mu {a}_{2,s}+{\rm{\Gamma }}{a}_{1,p}^{2}{a}_{1,i}^{\ast }\\ \frac{d{a}_{2,s}}{dt} & = & i({\omega }_{2,s}-{\omega }_{s}+\frac{i}{{\tau }_{{\rm{tot}}}}){a}_{2,s}-\mu {a}_{1,s}+{\rm{\Gamma }}{a}_{2,p}^{2}{a}_{2,i}^{\ast }\end{array}$$

where ${\tau }_{{\rm{tot}}}=\frac{{\tau }_{e}{\tau }_{i}}{{\tau }_{e}+2{\tau }_{i}}$ is the total photon lifetime, $\mu =\frac{2}{{\tau }_{e}}{e}^{-i\varphi }$ is the coupling constant and |Γ|^2^ is the rate of energy conversion into the Signal wave. In Equation 1, we neglect all the FWM terms except the one involved in Signal generation, as well as thermal and free carrier induced resonance shifts, since their effect will be negligible in the experiment described later. The amplitude of the fields in Equation 1 depend just on time, since all the spatial degrees of freedom are embedded into the effective nonlinear coupling term Γ^37^. This approximation holds since the roundtrip time of light inside the cavity (ps regime) is much shorter than the coherence time of the CW lasers (*μ*s regime), so that the field envelope can be considered spatially uniform inside the resonator. Within this approximation, the mean field equations shown in Equation 1 give the same conversion efficiency as it would be obtained by a more general treatment in which the field propagation inside the resonator is also taken into account^38^. Under the undepleted Pump (and Idler) approximation^39^, the Pump and Idler amplitudes *a*_1(2),*p*(*i*)_ are given by *a*_1(2),*p*(*i*)_ = *E*_1(2),*p*(*i*)_*p*_*p*(*i*)_, where:

2 $$\begin{array}{rcl}{E}_{\mathrm{1,}p(i)} & = & \tfrac{{\tau }_{{\rm{tot}}}({\tau }_{{\rm{tot}}}(i{\mu }^{2}\sqrt{{\tau }_{e}}-2{{\rm{\Delta }}}_{p(i)}\sqrt{\tfrac{1}{{\tau }_{e}}})-2i\sqrt{\tfrac{1}{{\tau }_{e}}})}{\sqrt{2}({\tau }_{{\rm{tot}}}^{2}(({{\rm{\Delta }}}_{p(i)}+{\delta }_{p(i)}){{\rm{\Delta }}}_{p(i)}+{\mu }^{2})+i(({\rm{\Delta }}+{\delta }_{p(i)})+{{\rm{\Delta }}}_{p(i)}){\tau }_{{\rm{tot}}}-1)}\\ {E}_{\mathrm{2,}p(i)} & = & \tfrac{i\mu {\tau }_{{\rm{tot}}}(2\sqrt{\tfrac{1}{{\tau }_{e}}}{\tau }_{{\rm{tot}}}+\sqrt{{\tau }_{e}}(i({\rm{\Delta }}+{\delta }_{p(i)}){\tau }_{{\rm{tot}}}-1))}{\sqrt{2}({\tau }_{{\rm{tot}}}^{2}(({\rm{\Delta }}+{\delta }_{p(i)}){{\rm{\Delta }}}_{p(i)}+{\mu }^{2})+i(({\rm{\Delta }}+{\delta }_{p(i)})+{{\rm{\Delta }}}_{p(i)}){\tau }_{{\rm{tot}}}-1)}\end{array}$$

are the Energy Enhancement factors (EE) for resonator 1 and 2, respectively, which have units of $\sqrt{{\rm{s}}}$. In Equation 2 the quantities Δ_*p*(*i*)_ = (*ω*_2,*p*(*i*)_ − *ω*_*p*(*i*)_) and *δ*_*p*(*i*)_ = (*ω*_1,*p*(*i*)_ − *ω*_2,*p*(*i*)_) have been introduced. To derive the expression of the generated Signal, we insert Equation 2 into the set of Equation 1, and we solve for *a*_1(2),*s*_, obtaining:

3 $$\begin{array}{rcl}{a}_{\mathrm{1,}s} & = & \tfrac{{\rm{\Gamma }}{p}_{p}^{2}{p}_{i}^{\ast }{\tau }_{{\rm{tot}}}(\mu {E}_{\mathrm{2,}p}^{2}{E}_{\mathrm{2,}i}^{\ast }{\tau }_{{\rm{tot}}}+{E}_{\mathrm{1,}p}^{2}{E}_{\mathrm{1,}i}^{\ast }(i{{\rm{\Delta }}}_{s}{\tau }_{{\rm{tot}}}-1))}{{\tau }_{{\rm{tot}}}^{2}(({{\rm{\Delta }}}_{s}+{\delta }_{s}){{\rm{\Delta }}}_{s}+{\mu }^{2})+i(({{\rm{\Delta }}}_{s}+{\delta }_{s})+{{\rm{\Delta }}}_{s}){\tau }_{{\rm{tot}}}-1}\\ {a}_{\mathrm{2,}s} & = & \tfrac{{\rm{\Gamma }}{p}_{p}^{2}{p}_{i}^{\ast }{\tau }_{{\rm{tot}}}(\mu {E}_{\mathrm{1,}p}^{2}{E}_{\mathrm{1,}i}^{\ast }{\tau }_{{\rm{tot}}}+{E}_{\mathrm{2,}p}^{2}{E}_{\mathrm{2,}s}^{\ast }(i({{\rm{\Delta }}}_{s}+{\delta }_{s}){\tau }_{{\rm{tot}}}-1))}{{\tau }_{{\rm{tot}}}^{2}(({{\rm{\Delta }}}_{s}+{\delta }_{s}){{\rm{\Delta }}}_{s}+{\mu }^{2})+i(({{\rm{\Delta }}}_{s}+{\delta }_{s})+{{\rm{\Delta }}}_{s}){\tau }_{{\rm{tot}}}-1}\end{array}$$

The Signal power in the drop port *P*_*s*_ is the coherent sum of the Signals generated by the two resonators and coupled into the output waveguide, and is given by ${P}_{s}=\frac{2}{{\tau }_{e}}|{a}_{1,s}+{e}^{-i\varphi ({\omega }_{s})}{a}_{2,s}{|}^{2}$.

By using Equation 3, this expression reduces to the simple form ${P}_{s}={{\rm{\Gamma }}}^{2}{P}_{p}^{2}{P}_{i}({\gamma }_{1}+{\gamma }_{2}+{\gamma }_{12})$, in which:

4 $$\begin{array}{rcl}{\gamma }_{1} & = & |{E}_{\mathrm{1,}p}{|}^{4}|{E}_{\mathrm{1,}i}{|}^{2}|{E}_{\mathrm{1,}s}{|}^{2}\\ {\gamma }_{2} & = & |{E}_{\mathrm{2,}p}{|}^{4}|{E}_{\mathrm{2,}i}{|}^{2}|{E}_{\mathrm{2,}s}{|}^{2}\\ {\gamma }_{12} & = & 2\Re ({E}_{\mathrm{1,}p}^{2}{({E}_{\mathrm{2,}p}^{\ast })}^{2}{E}_{\mathrm{1,}s}^{\ast }{E}_{\mathrm{2,}s}{E}_{\mathrm{1,}i}{E}_{\mathrm{2,}i}^{\ast })\end{array}$$

**Figure 2:**
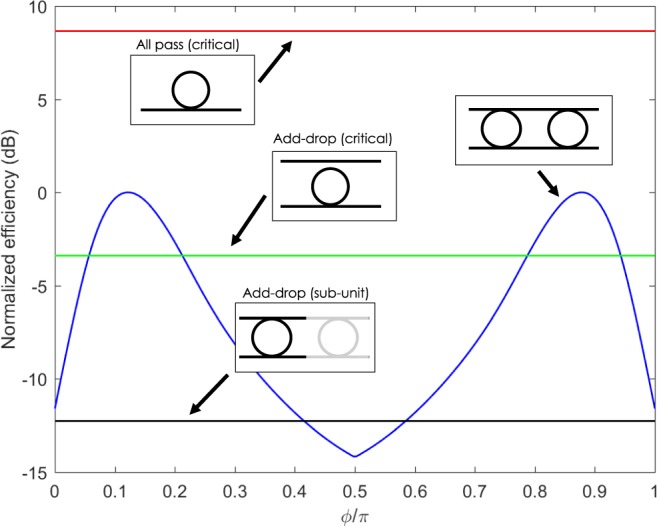
The FWM conversion efficiency of the photonic molecule (blue curve), as a function of the phase *ϕ*, is compared to the one of the critically coupled All-Pass (red curve) and Add-Drop (green curve) resonators, and to the one of the Add-Drop resonators which form the molecule (black curve). The maximum of the conversion efficiency of the photonic molecule has been set to zero, and all the curves are referred to this level.

In Equation 4, we identify the term *γ*_1_ (*γ*_2_) as the Signal generated by ring 1 (ring 2) and coupled into the drop port. Since the two are coherent, the term *γ*_12_ takes into account the interference between them. From Equation 2, it is evident that it is possible to change the relative phase between the two Signal waves by acting on the resonator detuning *δ*, the pump detuning Δ and the inter-resonator phase *ϕ* (for the Pump, Idler and Signal). In this way, constructive or destructive interference can be realized. One natural question which arises is whether by constructive interference one can overcome the overall FWM efficiency *P*_*s*_/*P*_*i*_ of a single resonator. To investigate this, for each value of *ϕ* we maximized *P*_*s*_ in the two dimensional parameter space spanned by (*δ*, Δ), and we compare this quantity to the FWM efficiency of several single resonator configurations. These include the critically coupled All-Pass (single bus) and Add-Drop (double bus) resonator, whose efficiencies scale respectively as $\propto {(\sqrt{\frac{{\tau }_{i}}{2}})}^{8}$ and $\propto {(\sqrt{\frac{{\tau }_{e}}{2}})}^{8}$, and the Add-Drop resonators which forms the molecule, in which the efficiency scales as ${(\sqrt{\frac{{\tau }_{e}}{2}}{\tau }_{{\rm{tot}}})}^{8}$. We fix the intrinsic and the extrinsic photon lifetimes *τ*_*i*_ and *τ*_*e*_ to be *τ*_*i*_ = 250 ps and *τ*_*e*_ = 75 ps, which, as we will see later, represent meaningful values for our experiment, and we assume for simplicity *δ*_*p*_ = *δ*_*i*_ and Δ_*p*_ = Δ_*i*_. This latter choice does not account for the presence of dispersion in our simulation, but helps to reduce the number of degrees of freedom. With these parameters, the total photon lifetime is *τ*_tot_ = 32.6 ps, which is associated to a cavity linewidth of ~0.08 nm. The comparison in shown in Fig. 2.

**Figure 3:**
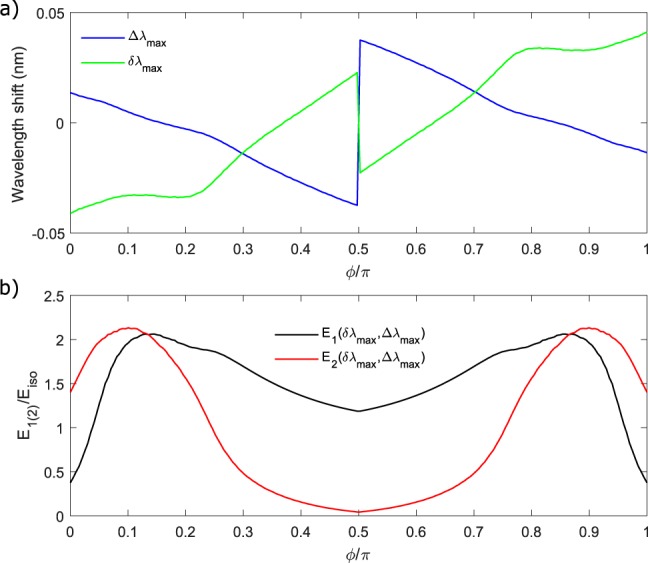
(**a**) Values of *δ*_max_ and Δ_max_ which maximizes the generation efficiency. (**b**) Energy enhancement factors E_1(2),*p*(*i*)_ of resonator 1 and 2 within the molecule normalized with respect to the one of the isolated resonator *E*_iso_. For each *ϕ*, these quantities are evaluated at the detunings *δ*_max_ and Δ_max_ which maximize the generation efficiency.

We notice the presence of two sweet spots, one at *ϕ* = 0.87*π* and the other at *ϕ* = 0.13*π*, in which the FWM efficiency of the molecule is maximized. When this occurs, the efficiency of the coupled resonator system is superior to the one of its internal constituents by approximately 12.5 dB, and overcomes also the one of a critically coupled Add-Drop resonator by 3.45 dB. However, the performance of the critically coupled All-Pass ring is never exceeded, being higher by 8.5 dB. This analysis indicates that the photonic molecule, while being superior within the class of the considered four port devices, still remains a sub-optimal configuration if compared to the single bus one. This could be intuitively understood from the fact that our coupling scheme employs two decay channels into the external waveguides, while the single bus configuration only one. The improved internal energy buildup, allowed by the inter-resonator coupling, is thus not sufficient to overcome the loss associated to the additional decay channel. However, as we will see later, even if the photonic molecule is an intrinsically sub-optimal configuration for reaching record FWM efficiencies, its internal degrees of freedom allows a level of coherent control of the FWM signal which can not be reached by single bus resonators. To complete our analysis, for each value of *ϕ*, we calculated the values of (*δ*_max_, Δ_max_) which maximizes the overall FWM efficiency. Since in our experiment is more pratical to deal with spectral detunings (*δλ*_max_, Δ*λ*_max_), in Fig. 3(a) we reported these quantities in place of (*δ*_max_, Δ_max_). Note that *δλ*_max_ is the resonant detuning while Δ*λ*_max_ is the pump detuning. We notice that in correspondence to the two sweet spots *ϕ* = 0.13*π* and *ϕ* = 0.87*π*, the resonances of the two rings are slightly detuned by an amount *δλ*_max_ = ±0.03 nm, while Δ*λ*_max_ ~ 0, meaning that the pump wavelength almost coincides with the resonance of the ring 2. The maximum FWM efficiency is thus realized in strongly asymmetric conditions, in which the two rings have different resonance wavelengths and only ring 2 is resonating with the Pump (and the stimulating Idler). From Fig. 3(b) we can track the internal energy enhancements |*E*_1(2),*p*(*i*)_(Δ_max_,*δ*_max_)|^2^ of the two rings, normalized to the EE of the same rings when they are not coupled (we refer to this condition as the *isolated* resonator, and denote the associated energy enhancement factor as *E*_iso_). At *ϕ* = 0.13*π* or *ϕ* = 0.87*π*, the energies of the resonators both overcome the one of the isolated cavity, a condition that we denote as Coupled Resonator Field Enhancement (CRFE). If no CRFE would occur, the EE factors in Equation 4 would be all lower than |*E*_iso_|, so that the maximum level of generated Signal will never exceed 4*P*_*s*,iso_, in which *P*_*s*,iso_ is the Signal generated for an isolated resonator coupled to the drop port. This simple check can be used in the experiment to distinguish whether the increase of the FWM efficiency is due to the coherent interference of the signal waves generated by the two resonators, or if it is due to the CRFE effect, without resorting to the probing of the individual internal energies.

### Device and experimental set-up

**Figure 4:**
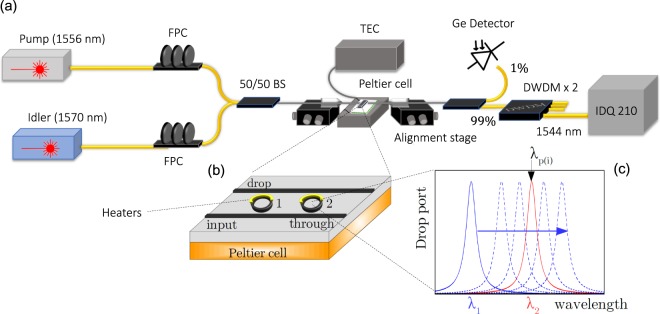
(**a**) Experimental set-up used for studying stimulated FWM. FPC = Fiber Polarization Controller, BS = Beam Splitter, DWDM = Dense Wavelength Division Multiplexing Module, TEC = Thermoelecric Controller. (**b**) Zoom of the device under test: the sample is in thermal contact with a Peltier Cell and on top of each resonator, thermal phase shifters (metallic heaters) are placed. (**c**) Schematic of the device operation: while the Pump (Idler) laser wavelength is resonantly coupled with a fixed ring 2 resonance (red curve), the ring 1 resonance (blue curve) is swept with the heater from a blue-shifted to a red-shifted configuration. The generated Signal through stimulated FWM is acquired at different temperatures of the Peltier cell, i.e. for different phases *ϕ* between the two resonators. Note that the Pump, Idler laser wavelengths are set to fulfill the FWM energy conservation, i.e to different ring 2 resonances.

The optical set-up is sketched in Fig. 4(a). Two C-band, tunable, CW lasers, are butt coupled in the input waveguide by means of tapered lensed fibers using nanometric positioning stages. As shown in Fig. 4(b), the device under test is composed by two ring resonators in the Add-Drop filter configuration. The waveguide cross section is 500 × 220 nm^2^, embedded in silica. The internal radii are *R*_1_ ~ *R*_2_ ~ 6.5 μm. The center to center distance between the two rings is *L* = 53.015 μm. Above each resonator, a micro-metallic heater is placed, sketched as a yellow half-moon in Fig. 4(b). In this way, it is possible to tune the resonance wavelength of each resonator through the thermo-optic effect. Moreover, the device is in thermal contact with a Peltier cell (sketched as the nethermost yellowish box in Fig. 4(b)), whose temperature can be controlled in the range 25–70 °C, with an accuracy of ±1 °C. This provides the control of the relative phase *ϕ* between the two resonators.

An infrared camera images the device surface. It has actually a twofold task: to easy the fiber alignment and to monitor the top-scattered light by the two resonators. The two input lasers provide the Pump (*λ*_*p*_) and the Idler (*λ*_*i*_) waves for the stimulated FWM process. Their wavelengths are tuned into two adjacent resonance orders of ring 2 in order to maximize the generated Signal. Note that, this condition may be slightly different from a perfectly resonant excitation, due to Fabry-Perot reflections at the end facets of the device. The choice of the resonant excitation of ring 2 is motivated by the analysis carried out in Fig. 3(a), in which the condition Δ*λ* ~ 0 has to be satisfied in order to observe the sweet spots. The Pump and Idler powers have been set to ~25 *μ*W (−16 dBm) (within the input waveguide), which allows to fully accommodate the dynamic range of the output Signal for every investigated configuration of the device. These values have been also raised up to 0.75 mW without noticing any thermal and free carrier effects, meaning that the conversion efficiency could be raised by three orders of magnitude just by increasing the pump power. The resonance wavelengths of ring 2 are adjusted by the micro-heater in a configuration such that the Signal wavelength fits within our Dense Wavelength Division Multiplexing (DWDM) module (*λ*_*s*_ = 1544 nm). This allows us performing an efficient filtering of the Pump and Idler to detect only the generated Signal photons. The output light signal is collected with a tapered fibre from the Drop port. 1% of it goes to a germanium detector in order to monitor the level of the total transmitted power, while the rest is directed to the filtering stage. Then, the Signal is separated from the co-propagating Pump and Idler by using two cascaded DWDM modules, and directed to a single photon counter.

The measurement proceeds as follows: starting from T = 25 °C, the Peltier temperature is progressively increased. The thermal red-shift of ring 2 resonances is compensated by changing the current which flows into the corresponding micro-heater. In this way, the ring 2 Signal resonance remains locked within the chosen DWDM channel regardless the Peltier temperature, and the net effect of the temperature increase is to change the value of *ϕ*. A temperature variation in the range 25–70 °C produces a phase variation in the range (0.36–0.91)*π* (more detals in the Methods section). Ring 1 resonance wavelengths are then swept over the ones of ring 2, thus changing the relative eigenfrequency detuning *δ*. An intuitive sketch of the measurement is reported in Fig. 4(c).

**Figure 5:**
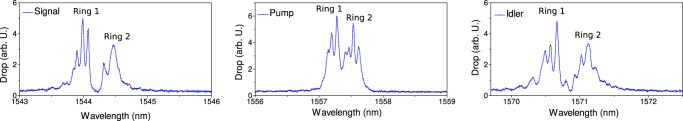
From left to right, Drop port spectra for Signal, Pump, Idler resonances of ring 1 and ring 2 measured at 33. Ring 1 is set to be blue-detuned from ring 2 of about Δ*λ* ~ 0.5 nm. Peltier temperature was set to 33 °C.

Figure 5 reports the light coupled into the Drop port in the spectral intervals which cover the Pump, the Signal and Idler resonances. The polarization has been set to Transverse Electric (TE).

The measure was taken with the Peltier set to 33 °C and with the two resonators having a relative detuning of 0.5 nm between their resonaces. From these spectra we estimate a free spectral range of about 13.5 nm and a total quality factor of about 19500. The main source of error in these estimations comes from the strong Fabry-Perot oscillations due to waveguide end facets reflections, which are superimposed on the micro-ring spectral response, as it can be seen from Fig. 5. We point out that these unwanted oscillations can be effectively suppressed by making use of grating couplers or inverse tapers.

It is possible to express the total quality factor in terms of the photon lifetime *τ*_tot_ through the relation *Q* = *ωτ*_tot_/2^40^. From this relation the total photon lifetime results to be about *τ*_tot_ = 32 ps.

### Stimulated Four Wave Mixing control in the photonic molecule

**Figure 6:**
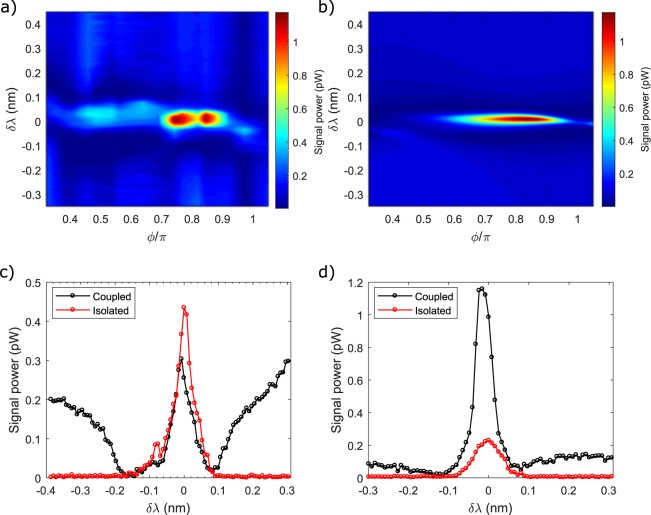
(**a**) Measured Signal power in the parameter space defined by the resonance wavelength detuning *δλ* between ring 1 and 2, and inter-resonator phase *ϕ*. The Pump and Idler wavelengths are kept fixed and locked to ring 2 resonances, and their power set to 25 *μ*W. (**b**) Fit of the Signal power in the same parameter space defined in (**a**). (**c**) Signal intensity at the not-enhancing phase *ϕ* = 0.45*π* as a function of *δλ* for the coupled (black) and the isolated (red, only ring 1 excited) configuration. (**d**) Same as in (**c**), but for the enhancing phase *ϕ* = 0.85*π*. The detuning *λ*_1_ − *λ*_*p*_ which appears in panels (c,d) refers to the isolated case, and represents the detuning of the resonance wavelength *λ*_1_ of ring 1 with respect to the Pump(Idler) wavelength *λ*_*p*_.

Stimulated FWM is investigated in the parameter space defined by the phase *ϕ* and by the detuning *δλ* = *λ*_1_ − *λ*_2_ between the resonance wavelengths *λ*_1(2)_ of the two resonators. The two-dimensional experimental map of the generated Signal power, as a function of *δλ* and *ϕ*, is reported in Fig. 6(a), while in Fig. 6(b) there is the simulated map according to Equation 4.

In Fig. 6(a,b), the power is referred to the output of the Drop waveguide, which has been obtained by measuring the off-chip power and by subtracting the total losses before the detector (13.5 dB) and by taking into account its detection efficiency. Different regions of the parameter space reveal distinct regimes. When the detuning *δλ* is greater than ~0.3 nm, which corresponds roughly to three times the resonator resonance linewidth, the two resonators become effectively decoupled. In this case, the generated Signal is essentially the one generated by ring 2. This is consistent with the fact that the Pump and Idler wavelengths are locked to the ring 2 resonances in our pumping scheme. Since the two resonators are decoupled, the inter-resonator phase *ϕ* has no effect in this region of the parameter space. In the rest of this paper, we will use the term *isolated* to refer to this configuration in which the relative detuning *δλ* greatly exceeds the linewidth of the individual resonator resonances. In order to compare the behaviour of the coupled configuration to the one of an isolated resonator, we tuned the resonance wavelength of ring 2 very far from the Pump(Idler) wavelength (about 2 nm), so that the system reduces to a single resonator where only ring 1 is effective. In this case, the detuning *δλ* which appears in Fig. 6(c,d) for the isolated case has to be interpreted as the detuning of *λ*_1_ with respect to the Pump(Idler) wavelength.

As $|\delta \lambda |\to 0$, the resonator coupling gets important, and the generated Signal strongly depends on the value of *ϕ*. In the broad interval defined by 0.3*π* < *ϕ* < 0.7*π*, the dependence on *ϕ* is weak. Figure 6(c) shows a detail of the Signal behaviour at *ϕ* = 0.45*π*. When the two resonators are slightly detuned ($|\delta \lambda |\lesssim 0.1$ nm), we observe a complete suppression of the Signal. This suppression ends when the resonance wavelengths of the two resonators are almost overlapped (*δλ* ~ 0). In this case, the generated Signal peaks. As it is clear from Fig. 6(c), the Signal intensity of the coupled system never exceeds the one of the isolated resonator, as expected also from the simulation.

Very interestingly, in the interval defined by 0.7*π* < *ϕ* < 0.9*π*, Signal suppression is still observed for $|\delta \lambda |\lesssim 0.1$ nm. On the contrary, when the two resonator resonances become almost overlapped, a clear enhancement of the conversion efficiency with respect to the isolated resonator Signal occurs. This is more evident in Fig. 6(d), which shows a slice of the two-dimensional map of Fig. 6(a) at *ϕ* = 0.85*π*. Here, the enhancement with respect to the FWM efficiency of the isolated resonator is (7.0 ± 0.2) dB. Since this value corresponds to an enhancement of more than a factor of 4, it has to be necessarily attributed to the CRFE effect. The observation of a sweet spot in correspondance to *ϕ* = 0.85*π* is also in quite good agreement with the theoretical preditions shown in Fig. 2. We emphasize that even in the best configuration, the conversion efficieny is (−72.4 ± 0.2) dB, which is three orders of magnitude lower compared to other related works^17,20,41^. However, we stress that our aim is to show the possibility to coherently control FWM, not to demonstrate record efficiencies. As already discussed above, the conversion efficiency could be improved by increasing the pump power without compromising the coherent control. As an example, a conversion efficiency of −45 dB could be achieved by raising the pump power to 0.5 mW.

In order to validate the experimental results, we fit the experimental data by using Equation 4 and by fully taking into account the eigenfrequency dispersion as a function of wavelength. With reference to Equation 2, we let (*L*, *P*_*p*_, *τ*_*e*_, Δ_*p*_, Δ_*i*_) to be free parameters (more details are provided in the Methods section). The result of the fit is shown in Fig. 6(b). A general qualitative agreement is found between theory and experiment. The best matching between the simulation and the experiment is found by setting the input Pump and Idler wavelengths slightly red detuned (0.03 nm and 0.013 nm respectively) with respect to the corresponding resonance wavelengths of ring 2, and the pump power to 8.4 *μ*W. The lower input Pump power compared to the experiment could be attribuited to an underestimation of the loss. We already commented that the wavelength mismatch is mainly due to the Fabry-Perot reflections at the input-output facets of the sample. We point out that also the double enhancement spots at *ϕ* = 0.75*π* and *ϕ* = 0.85*π* in Fig. 6(a) are due to these reflections which affect the Pump and Idler intensities. Indeed, as suggested by the fit in Fig. 6(b), the maximum enhancing phase should be unique and placed approximately at *ϕ* = 0.8*π*. We excluded the possibility that the observed enhancement is due to the coherent interference of these reflections by averaging the Signal generated by the isolated resonator over multiple temperatures. The same was done for the Signal recorded in the coupled configuration by varying the temperature across the enhancement point. We did not include the effect of these reflections in Equation 1 since already in its simplified form it is capable to grasp all the observed interference phenomena. The matching with the experiment should then be considered as only qualitative.

**Figure 7:**
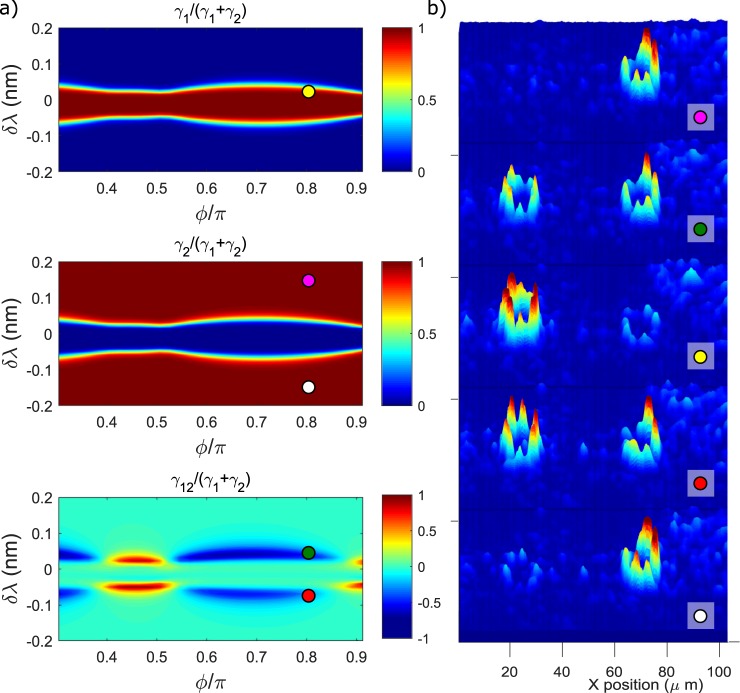
(**a**) From top to bottom: Signal generated by the resonator 1 (*γ*_1_ in Equation 4), Signal generated by the resonator 2 (*γ*_2_ in Equation 4) and effect of mutual interference (*γ*_12_ in Equation 4). The coloured dots refer to the points in the parameter space defined by *δλ* and *ϕ* where the top scattering patterns in panel (b) have been measured. (**b**) Top images of the light scattered by the two resonators at different detunings *δλ* and for a fixed value of the phase *ϕ* = 0.8*π* (enhancing phase for the simulation). The leftmost resonator corresponds to ring 1. Peaks and valleys in the scattering patterns are due to inhomogeneities of the scattering centres. The maximum intensity is indicated with a red color, while the minimum intensity with a blue color.

**Figure 8:**
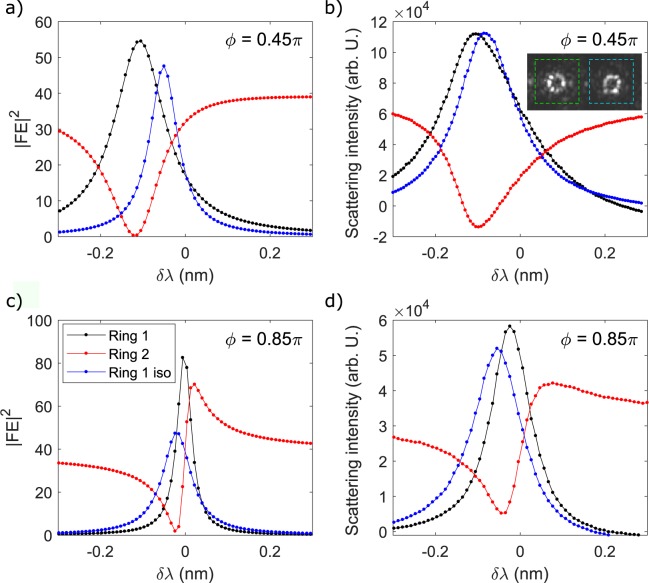
(**a**) Simulated internal field enhancement |*FE*|^2^ of ring 1 (black dotted line) and ring 2 (red dotted line) in the coupled configuration. The field enhancement of ring 1 when it is isolated from ring 2 is shown by the blue dotted line. Here, the detuning Δ*λ* in the isolated configuration has the same meaning as in Fig. 6(c,d). This simulation assumes *ϕ* = 0.45*π*. (**b**) Top scattering intensity integrated over ring 1 and 2 when *ϕ* = 0.45*π*. The intensity relative to ring 2 near zero detuning is negative because of the background subtraction. The inset shows an example of the top scattering pattern imaged by our camera. The green and the cyan dashed boxes enclose the two areas where the pixel intensities are integrated to obtain the black and red dotted curves respectively. (**c**) Same as in (**a**) for *ϕ* = 0.85*π*. (**d**) Same as in (**b**) for *ϕ* = 0.85*π*. In all panels the field enhancement and the integrated top scattering refers to the Pump wave. In panels (b–d) errorbars are smaller than the size of the scatters used to plot the data.

Having validated the simulation, we can look more carefully at the physics underlying the different regimes in Fig. 6. The contributions of the two resonators *γ*_1_ and *γ*_2_, and of their interference *γ*_12_ (see Equation 4) to the total dropped Signal intensity is shown Fig. 7(a). At large detunings (indicated with magenta and white dots in Fig. 7(a)), only ring 2 is excited, and FWM only occurs within this resonator, i.e., ${\gamma }_{2}/({\gamma }_{1}+{\gamma }_{2})\to 1$. As $\delta \lambda \to 0$, the field inside ring 2 is progressively decreased, while the one of ring 1 grows. At the transition points, when the energy is equally shared between the two resonators, the Signal waves generated by FWM have equal amplitudes but they can be out of phase, giving raise to destructive interference in the drop channel (green and red dots in Fig. 7(a)). This is the origin of the FWM suppression observed in Fig. 6(d). Intriguingly, from Fig. 7(a), we observe that at *ϕ* = 0.45*π*, the interference between the two Signal waves is constructive, but in Fig. 6(c) we still observe Signal suppression with respect to large detunings *δλ*, i.e., when only ring 2 is active. This ambiguity is solved by looking at the simulation shown in Fig. 6(b). Here, we see that at *ϕ* = 0.45*π*, the FWM signal is always very low when the two resonators are coupled ($|\delta \lambda |\lesssim 0.1\,{\rm{nm}}$), meaning that the fields inside both rings are suppressed by mutual interference. Thus, even if the interference of the Signal waves is constructive, the overall intensity at $|\delta \lambda |\lesssim 0.1\,{\rm{nm}}$ is still lower than the one at large detunings, thus appearing in Fig. 6(c) as an effective suppression of the Signal. At the enhancement point, indicated with a yellow dot in Fig. 7, the energy is mostly localized in ring 1. Even if this feature contrasts with the energy distributions at the sweet spots shown in Fig. 3(b) (in which the two internal energies are predicted to be almost equal), we will see later that it well matches the experimental results. The discrepancy should be attributed to the fact that the results of Fig. 3(b) have been obtained using a simplistic model which neglects dispersion and assumes a perfect resonant excitation of ring 2. The inclusion of off-resonant excitation and dispersion is instead fully accounted in the model used to compute the conversion efficiency depicted in Fig. 6(b). We recorded the top scattering images of the two resonators as a function of the detuning *δλ* for different phases, one corresponding to the enhancement point *ϕ* = 0.85*π*, one to *ϕ* = 0.45*π*, and one at the enhancing phase of the simulation at *ϕ* = 0.8*π*. Some of these scattering patterns, corresponding to the values of *δλ* indicated by the coloured dots in Fig. 7(a), are shown in Fig. 7(b). The total integrated scattering intensities as a function of *δλ* are shown in Fig. 8. Remarkably, we see from Fig. 7(b), that the field distribution between the two resonators closely follows the theoretical predictions shown in Fig. 7(a). In particular, when Signal suppression is observed, both resonators appear equally bright, and, when Signal enhancement occurs, the fields are found mostly localized in ring 1. The comparison between the simulated field enhancements in Fig. 8(a,c) is also in qualitative agreement with the measured ones, indicated in Fig. 8(b,d). It is clear from Fig. 8(c), that at the experimental enhancing phase *ϕ* = 0.85*π*, the maximum energy stored inside ring 1 in the coupled configuration is almost twice the one of the isolated configuration. This is due to the CRFE effect. At *ϕ* = 0.45*π*, this effect is much weaker. This is also observed from the experimental curves shown in Fig. 8(b,d). At *ϕ* = 0.85*π* and ${\rm{\Delta }}\lambda \simeq 0$, ring 1 is coupled to ring 2 and appears to be brighter than the isolated ring. Here the difference in brightness is less remarked than in the simulation. This is an experimental limitation of the measurement due to the poor dynamic range of the image sensor. At *ϕ* = 0.45*π*, CRFE is not effective, and we cannot resolve the difference between the coupled and the isolated configuration.

### Supermode analysis of coupled resonators

An alternative approach to solve the set of Equation 1 for the Pump and the Idler energy amplitudes *a*_1(2),*p*(*i*)_ is to make a change of basis such that the equations of motion become uncoupled. This corresponds to consider the system as a whole and to determine the eigen-modes supported by the photonic molecule.

We rewrite the equation for the Pump (or the Idler) amplitude *a* using the following compact matrix notation:

5 $${\mathbb{M}}\bar{a}-i{\omega }_{p}1+\bar{b}=0$$

where

$${\mathbb{M}}=[\begin{array}{cc}i{\omega }_{1}-\frac{1}{{\tau }_{{\rm{tot}}}} & -\mu \\ -\mu  & i{\omega }_{2}-\frac{1}{{\tau }_{{\rm{tot}}}}\end{array}]\,{\mathbb{1}}=[\begin{array}{cc}1 & 0\\ 0 & 1\end{array}]$$

and

$$\bar{a}=[\begin{array}{c}{\bar{a}}_{1}\\ {\bar{a}}_{2}\end{array}]\,\bar{b}=[\begin{array}{c}i\sqrt{\frac{2}{{\tau }_{{\rm{e}}}}}\\ i\sqrt{\frac{2}{{\tau }_{{\rm{e}}}}}\,{e}^{-i\varphi }\end{array}]$$

For simplicity we have set the input power equal to one. We now look for a change of coordinates $\bar{a}\to {\bar{a}}_{s}$ described by $\bar{a}={\mathbb{P}}{\bar{a}}_{s}$, such that ${{\mathbb{P}}}^{-1}\,{\mathbb{M}}{\mathbb{P}}$ is diagonal. The matrix ${\mathbb{P}}$ will then have on its columns the eigenvectors of ${\mathbb{M}}$, and the diagonal elements of ${{\mathbb{P}}}^{-1}\,{\mathbb{M}}{\mathbb{P}}$ will be the eigenvalues of ${\mathbb{P}}$. By solving the characteristic equation, the eigenvalues *η*_1,2_ are given by:

6 $${\eta }_{\mathrm{1,2}}=-\,\frac{1}{{\tau }_{{\rm{tot}}}}+\frac{i({\omega }_{1}+{\omega }_{2})}{2}\pm \frac{1}{2}\sqrt{\frac{16}{{\tau }_{e}^{2}}(\cos \,2\varphi -i\,\sin \,2\varphi )-{\delta }^{2}}$$

where *δ* = *ω*_1_ − *ω*_2_ is the eigenfrequency detuning between the two resonators. The matrix ${\mathbb{P}}$ has the following form:

7 $${\mathbb{P}}=[\begin{array}{cc}\frac{\sqrt{4{\mu }^{2}-{\delta }^{2}}-i\delta }{2\mu } & \frac{-\,\sqrt{4{\mu }^{2}-{\delta }^{2}}-i\delta }{2\mu }\\ 1 & 1\end{array}]$$

while its inverse ${{\mathbb{P}}}^{-1}$ is given by:

8 $${{\mathbb{P}}}^{-1}=\frac{1}{{\rm{\det }}({\mathbb{P}})}[\begin{array}{cc}1 & \frac{\sqrt{4{\mu }^{2}-{\delta }^{2}}+i\delta }{2\mu }\\ -\,1 & \frac{\sqrt{4{\mu }^{2}-{\delta }^{2}}-i\delta }{2\mu }\end{array}]$$

The real part of Equation 6 gives the photon lifetimes *τ*_*s*1,*s*2_ of the supermodes of the structure, while the imaginary part represents their eigenfrequencies *ω*_*s*1,*s*2_. The supermode energy amplitudes *a*_*s*1_ and *a*_*s*2_ are given by:

9 $${a}_{s1}=\frac{-i\sqrt{\frac{2}{{\tau }_{e,s1}^{{\rm{down}}}}}}{i({\omega }_{s1}-{\omega }_{p})-\mathrm{1/}{\tau }_{s1}}$$

10 $${a}_{s2}=\frac{-i\sqrt{\frac{2}{{\tau }_{e,s2}^{{\rm{down}}}}}}{i({\omega }_{s2}-{\omega }_{p})-\mathrm{1/}{\tau }_{s2}}$$

where we have defined ${\tau }_{e,s1}^{{\rm{down}}}={\tau }_{e}/({P}_{11}^{-1}+{P}_{12}^{-1}\,{e}^{-i\varphi }{)}^{2}$ and ${\tau }_{e,s2}^{{\rm{down}}}={\tau }_{e}/({P}_{21}^{-1}+{P}_{22}^{-1}\,{e}^{-i\varphi }{))}^{2}$.

The complex power amplitude coupled into the drop port is given by:

11 $$\begin{array}{rcl}{p}_{{\rm{drop}}} & = & i\sqrt{\frac{2}{{\tau }_{e}}}{a}_{1}+i\sqrt{\frac{2}{{\tau }_{e}}}\,{e}^{-i\varphi }{a}_{2}\\  & = & [\frac{\sqrt{\frac{2}{{\tau }_{e,s1}^{{\rm{down}}}}}\sqrt{\frac{2}{{\tau }_{e,s1}^{{\rm{up}}}}}}{i({\omega }_{s1}-{\omega }_{p})-\mathrm{1/}{\tau }_{s1}}+\frac{\sqrt{\frac{2}{{\tau }_{e,s2}^{{\rm{down}}}}}\sqrt{\frac{2}{{\tau }_{e,s2}^{{\rm{up}}}}}}{i({\omega }_{s2}-{\omega }_{p})-\mathrm{1/}{\tau }_{s2}}]\end{array}$$

where we have defined ${\tau }_{e,s1}^{{\rm{up}}}={\tau }_{e}/({P}_{11}+{P}_{21}\,{e}^{-i\varphi }{)}^{2}$ and ${\tau }_{e,s2}^{{\rm{up}}}={\tau }_{e}/({P}_{12}+{P}_{22}\,{e}^{-i\varphi }{)}^{2}$.

From Equation 11 it is possible to recognize that the system composed by two symmetrical side-coupled ring resonators can be equivalently described by a single photonic molecule sustaining two uncoupled modes *a*_*s*1(2)_, which are *asymmetrically* coupled to the two bus waveguides. Therefore, even though the symmetric coupling of the two rings inherently forbids the critical coupling condition in presence of loss, the supermodes supported by the system do show an effective asymmetric coupling, hence they could be critically coupled by some combinations of the parameters *δ* and *ϕ*.

Some considerations about the validity of the supermode description of the system need to be discussed. The main stringent requirement is that the matrix ${\mathbb{P}}$ should be invertible, i.e., ${\rm{\det }}({\mathbb{P}})=\frac{{\tau }_{e}}{2}{e}^{i\varphi }\sqrt{\mathrm{16/}{\tau }_{e}^{2}{e}^{-i2\varphi }-{\delta }^{2}}\ne 0$. This is clearly not satisfied if ${\delta }^{2}\to \mathrm{16/}{\tau }_{e}^{2}$ and *ϕ* = *mπ* (where *m* is an integer), for which we have that ${\tau }_{e,s1}^{{\rm{down}}}$, ${\tau }_{e,s2}^{{\rm{down}}}\to 0$. As we approach to this condition, the weak coupling approximation, which underneaths the validity of the coupled mode equations, ${\tau }_{e,s\mathrm{1(2)}}^{{\rm{down}}}\gg 2{\tau }_{rt}$ (*τ*_*rt*_ is the roundtrip time of light in the cavity), does not hold any more. It is worth to note that in the strong coupling regime, the inadequacy of Equations 9 and 10 is reflected in the violation of energy conservation. The extreme condition $\delta =\frac{4}{{\tau }_{e}}$ corresponds to the onset of Coupled Resonator Induced Transparency (CRIT) in the structure, since the supermode lifetimes become equal, and their eigenfrequecies start to symmetrically split with respect to the center frequency (*ω*_1_ + *ω*_2_)/2^27^.

**Figure 9:**
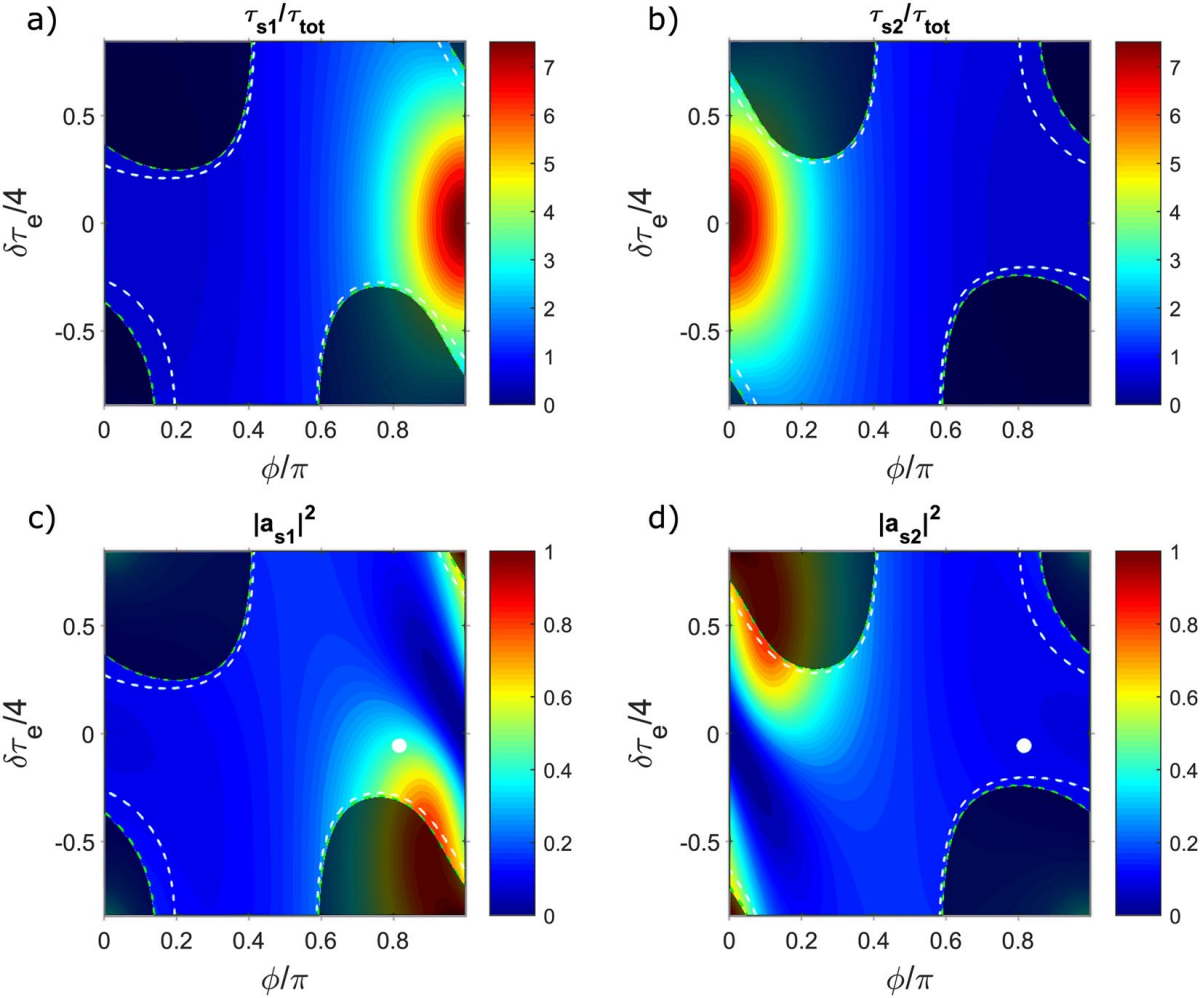
(**a**) Lifetime *τ*_*s*1_ of supermode 1 normalized with respect to the lifetime of of the isolated resonator *τ*_tot_. (**b**) Lifetime *τ*_*s*2_ of supermode 2 normalized with respect to the lifetime of the of the isolated resonator *τ*_tot_. (**c**) Normalized energy carried by supermode 1 when excited with a laser at frequency *ω*_*p*_ (we used the same value of *ω*_*p*_ used in the simulation shown in Fig. 6(b)). (**d**) Normalized energy carried by supermode 2 when excited with a laser at frequency *ω*_*p*_. In all the panels, the dashed white lines indicate where the respective supermodes are critically coupled, while the shaded regions indicate where energy conservation is violated due to the loss of the weak coupling regime. The white dots in panels (c and d) are placed where Signal enhancement was observed in experiments (see Fig. 6(a,d)).

Figure 9(a,b) shows the photon lifetimes of the two supermodes, which have been normalized with respect to the total photon lifetime of the isolated resonator *τ*_tot_. The green contour lines are placed where the quantity $\mathrm{1/}{\tau }_{s\mathrm{1(2)}}-\mathrm{1/}{\tau }_{e,s\mathrm{1(2)}}^{{\rm{up}}}-\mathrm{1/}{\tau }_{e,s\mathrm{1(2)}}^{{\rm{down}}}=0$, i.e., they enclose the regions where energy conservation is violated (dark-shaded zones in Fig. 9). Within these regions, $\mathrm{1/}{\tau }_{s\mathrm{1(2)}}-\mathrm{1/}{\tau }_{e,s\mathrm{1(2)}}^{{\rm{up}}}-\mathrm{1/}{\tau }_{e,s\mathrm{1(2)}}^{{\rm{down}}} < 0$, so they have no physical meaning. One can see that at *ϕ* = *π* and *δ* = 0, the supermode 1 has the maximum lifetime, corresponding to *τ*_*s*1_ = *τ*_*i*_. On the contrary, at *ϕ* = 0 and *δ* = 0, its photon lifetime is minimized. Supermode 2 has instead the opposite behaviour. Experimentally, the condition *ϕ* = 0,*π* could be reached at different temperatures of the Peltier cell depending on the value of the inter-resonator separation *L*, since $\varphi ={{\rm{mod}}}_{2\pi }({\varphi }_{0}(L)+{\rm{\Delta }}\varphi ({\rm{\Delta }}T))$, where ${\varphi }_{0}=\frac{\omega {n}_{{\rm{neff}}}L}{c}$ and Δ*ϕ*(Δ*T*) is defined above in the text. The energy stored by the two supermodes |*a*_*s*1(2)_|^2^, evaluated at the Pump frequency *ω*_*p*_ of the experiment, is shown in Fig. 9(c,d). At the coordinate point in the parameter space (*ϕ*,*δ*) where Signal enhancement is experimentally observed (white dot in Fig. 9(c,d)), we can see that the total energy is essentially carried by supermode 1. This is a consequence of its increased photon lifetime. Since $\bar{a}={\mathbb{P}}{\bar{a}}_{s}$, the high-Q factor of this supermode is shared by the two resonators, which means that they become sub-radiant^29^. This is at the origin of the CRFE effect. As it can be noticed from Fig. 9(c,d), the highest energy is not localized where the supermode 1 shows the maximum sub-radiance. This is due to the fact that when *ϕ* = *π* and *δ* → 0, from Equation 8 we have that ${\tau }_{e,s1}^{{\rm{down}}}\to \infty$ (the same occurs for ${\tau }_{e,s1}^{{\rm{up}}}$), which is equivalent to say that the supermode becomes completely decoupled from any external excitation. Consequently, as experimentally observed, the point where FWM is maximized is located near the point of maximal sub-radiance, in a position determined by the Pump(Idler) frequency and waveguide dispersion. Indeed, at a fixed Peltier temperature and heater current, the phase *ϕ* and the detuning *δ* are different for the Pump, the Signal and the Idler field, since these quantities are all wavelength dependent. In general, the maximum of the field enhancement for these three waves will be reached in three different points of the parameter space (all near the maximal sub-radiance), and as shown by Equation 4, the overall enhancement of the FWM process is where their product is maximized.

**Figure 10:**
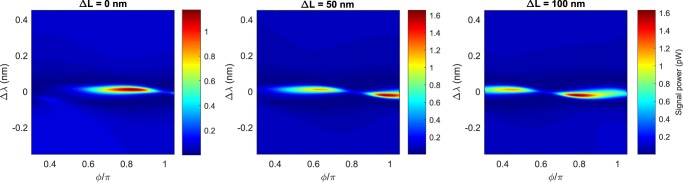
Generated Signal power in the photonic molecule for different values of the resonator separation *L* = *L*_0_ + Δ*L*, where *L*_0_ = 53.09 μm refers to the center to center distance between the two microring resonators determined by the fit of the experimental data shown in Fig. 6(a).

Finally, in Fig. 10 the impact of a small displacement in the inter-resonator separation *L* on the generated Signal is reported. The simulation uses the same parameters of the fit shown in Fig. 6(b), except from the parameter L which is swept in discrete steps Δ*L* around *L*_0_ = 53.09 μm, i.e., the value determined from the fit of the experiment. As expected, the phases at which the sweet spots associated to the two supermodes emerge depend on the value of L. There is a monotonic shift toward lower phases as the inter-resonator separation increases, with a period in L of approximately 300 nm. The resonator separation is then an additional degree of freedom for the FWM control in the photonic molecule.

## Discussion

In this article we presented, both theoretically and experimentally, a novel method to actively control the FWM interaction in a photonic molecule made by two side coupled silicon microring resonators. The tuning of the internal degrees of freedom of the molecule, e.g. the inter-resonator phase and their eigenfrequency detuning, allows a superior level of coherent control of the FWM output with respect to single resonators. Among the explored regimes, we found an enhancement of the Idler conversion efficiency of about a factor of 5 with respect to the maximum attainable level from each single constituent of the molecule, and attributed to the excitation of a sub-radiant mode. We call this phenomenon Coupled Resonator Field Enhancement, since the increase of the internal field of one of the two resonator is due to a coherent interaction with the other and vice-versa. CRFE fundamentally differs from a simple constructive interference between the FWM waves generated by the two resonators, since it necessarily requires the mutual coupling between the cavities to occur. In addition, we show that by controlling the inter-resonator phase, it is also possible to induce a suppression of the generated Signal waves. The coherent switching between those bright and dark states could be potentially implemented for signal processing. Carrier concentration modulation within and between the rings could be implemented (for example by means of pn junctions) in place of thermal tuning to achieve faster operational speeds. As a last remark, the rules derived within this manuscript could be naturally extended to *N* resonators. Indeed, a general consideration is that the number of supermodes scales with the number of microrings, so we should expect to observe 2*N* enhancing spots in a molecule made by *N* resonators. The number of degrees of freedom scales as 3(*N* − 1), since for each resonator that we add, we introduce the possibility to tune the phase of two more coupling waveguides and one eigenfrequency. There is no reason to believe that new FWM regimes will be introduced by increasing the number of resonators, since the overall power scattered into the Drop and Through ports will be always the coherent sum of all the waves scattered by each individual resonator. The Signal intensity in the 3(*N* − 1) dimensional phase space will be characterized by a complex pattern of enhanced and suppressed FWM regions similar to the ones observed for *N* = 2. It is worth to note that in the regime where $N\gg 1$, the maximum Signal intensity could overcome the one of an isolated, critically coupled All-Pass filter. As *N* increases, the system will start to behave like a photonic crystal made by coupled resonator, and the enhancement of the nonlinearity made by slow light will overcome the additional loss induced by the coupling with the Drop bus waveguide.

## Methods

### Device fabrication and experimental setup

The fabrication of the sample was carried out on a Silicon On Insulator (SOI) wafer, in which the waveguides have been patterned by 193 nm Deep UltraViolet (DUV) lithography. The waveguide has a width of 500 nm, an height of 220 nm, while the buried oxide has a thickness of 2 μm. The waveguides are covered by a 750 nm thick silica upper cladding. The internal radii are respectively, *R*_1_ = 6.495 μm, *R*_2_ = 6.505 μm. The center to center distance between the two rings is *L* = 53.015 μm, whereas the total length of the sample is 0.45 cm. The bus waveguide to ring gap is 160 nm, equal for both the bus waveguides. The waveguide is single mode for the Transverse Electric (TE) polarization at the wavelength of 1550 nm. Taking these geometrical parameters, the simulated external lifetime is *τ*_*e*_ = 75 ps, while by considering the measured value of 5 dBcm^−1^ for the linear propagation losses, the intrinsic lifetime is *τ*_*i*_ = 250 ps. Therefore, the ring resonators are excited in an *under-coupled* regime. The free spectral range of both the resonators is about 13.5 nm, and the quality factor is about *Q* = 19500. The waveguide presents a direct tapering from a waveguide width of 2 μm to the nominal waveguide width of 0.5 μm in 0.6 mm. Light is coupled in and out with tapered fibers in Butt coupling. The experienced 7 dB of coupling loss is consistent with the overlap integral calculation between the waveguide mode profile and the Gaussian spot size of the tapered lensed fibers (OZ optics). Titanium Nitride (TiN) micro heaters of width 400 nm, thickness 110 nm and and sheet resistance 14.56Ω/sq are placed on the top of the rings to locally change the refractive index through the thermo-optic effect. The generated FWM Signal photons inside the resonator are filtered from the co-propagating Pump and Idler beams by using two cascaded DWDM modules (Opneti) with an associated total insertion loss of ~7 dB. Signal photons are detected with a photon counter (ID Quantique ID210) operating in Free Running mode, with a detection efficiency of 5% and a dead time of 40 μs. The DWMD modules achieve a signal isolation of more than 100 dB.

### Fit of the experiment

The experimental data shown in Fig. 6(a) have been fit using Equation 4 and by letting ***x*** = (*L*, *P*_*p*_, *τ*_*e*_, Δ_*p*_, Δ_*i*_) as a vector of free parameters. We implemented a nonlinear optimization algorithm to minimize the sum of the square of the residuals *r*, and we used as a starting guess for ***x*** the values taken from the experiment. The optimization terminates when |*r*_*i*+1_ − *r*_*i*_| < 10^−6^, where *r*_*i*+1_ and *r*_*i*_ are the sums of the square of the residuals at iteration *i* + *i* and *i* respectively. The value of the fitted parameters, given with 95% of confidence bounds, are *L* = (53.0900 ± 0.0015) *μ*m, *P*_*p*_ = (8.4 ± 0.1) *μ*W, *τ*_*e*_ = (75.02 ± 0.018) ps, Δ_*p*_ = (3.00 ± 0.08) × 10^−2^ nm, Δ_*i*_ = (0.013 ± 0.001) nm. We fixed *τ*_*i*_ = 250 ps, *P*_*i*_ = 18 *μ*W and ${\rm{\Gamma }}=7.94\times {10}^{-8}\,\frac{ps}{mW\mu m}$. The fit returns a coefficient of determination of *R*^2^ = 0.304. Such a low value is mainly attributed to the fact that the model does not include the reflections at the input/output facets of the sample. Furthermore, a direct look at Fig. 6(a,b) reveals that the experimental enhancing spot is broadened along the vertical axis compared to the simulation. This probably arises from the electrical noise $(3.5\,\frac{\mu A}{\sqrt{Hz}})$ associated to the current which flows into the heater on ring 1. Indeed, to span the vertical axis, we changed the current in the interval ~[1.55–1.71] mA in steps of ~1.3 *μ*A, using for each step an integration time of 0.5 s. Since the current step is lower than the electrical noise, the relation between *δλ* and the heater current gets convoluted by the finite extension of the electrical point spread function (PSF) of the system, which has not been accounted in the model. We choose to not refine the model further since the inclusion of the reflections and of the system’s PSF would not change the underlying physics of the device.

### Phase variation induced by the Peltier

In order to map the change of the Peltier temperature Δ*T* into a phase variation *ϕ* = *ϕ*_0_ + Δ*ϕ*(Δ*T*), in which *ϕ*_0_ = *ϕ*(*T* = 25°), we recorded the spectral response of the device at different temperature steps. At each step, we monitored the shift Δ*λ*_1_ of the resonance of ring 1, and extracted the associated effective index change Δ*n*_eff_ as ${\rm{\Delta }}{n}_{{\rm{eff}}}({\rm{\Delta }}T)=\frac{{n}_{{\rm{eff}}}{\rm{\Delta }}{\lambda }_{1}({\rm{\Delta }}T)}{{\lambda }_{1}}$, where *λ*_1_ is the resonance wavelength at each step. The value of *n*_eff_ is calculated using a commercial Finite Element Method software. The same effective index change occurs also in the straight waveguides connecting the two resonators, since the whole chip is in thermal contact with the Peltier cell. The phase change induced in these sections is then ${\rm{\Delta }}\varphi ({\rm{\Delta }}T)=\frac{2\pi {\rm{\Delta }}{n}_{{\rm{eff}}}({\rm{\Delta }}T)L}{\lambda }$. A temperature variation in the range 25–70 produces a phase variation in the range (0.36–0.91)*π*. The lower temperature limit of 25 is set by the thermal capacity of the used Peltier cell, while the upper temperature limit of 70 is actually fixed by thermal instabilities of the whole chip observed at high temperature.
